# Prediction of Response in Head and Neck Tumor: Focus on Main Hot Topics in Research

**DOI:** 10.3389/fonc.2020.604965

**Published:** 2021-01-08

**Authors:** Liliana Belgioia, Silvia Daniela Morbelli, Renzo Corvò

**Affiliations:** ^1^ Radiation Oncology Department, IRCCS Ospedale Policlinico San Martino, Genoa, Italy; ^2^ Health Science Department (DISSAL), University of Genoa, Genoa, Italy; ^3^ Nuclear Medicine Department, IRCCS Ospedale Policlinico San Martino, Genoa, Italy

**Keywords:** head and neck cancer, radiation therapy, biomarkers, prognostic factors, predictive factors, precision medicine

## Abstract

Radiation therapy is a cornerstone in the treatment of head and neck cancer patients; actually, their management is based on clinical and radiological staging with all patients at the same stage treated in the same way. Recently the increasing knowledge in molecular characterization of head and neck cancer opens the way for a more tailored treatment. Patient outcomes could be improved by a personalized radiotherapy beyond technological and anatomical precision. Several tumor markers are under evaluation to understand their possible prognostic or predictive value. In this paper we discuss those markers specific for evaluate response to radiation therapy in head and neck cancer for a shift toward a biological personalization of radiotherapy.

## Introduction

Squamous cell carcinoma of the head and neck (SCCHN) accounts for about 4% of all malignant disease in adults. According to SEER data the 5-year OS is approximately 60% (1). Radiation therapy is a cornerstone in the treatment of these patients, and prognosis is influenced by several clinical factors as disease stage, site, HPV/EBV positivity, age and co-morbidity. About 50% of patients with head and neck cancer (HNC) present a locally advanced stage at diagnosis and are treated with multimodality therapy but the prognosis of these patients is still not satisfactory (2).

The evolution of treatments in oncology is moving towards precision medicine that is the cross from the so-called “one-size-fit-all medicine” to stratified treatments on certain subpopulations of patients based, for example, on disease subtypes, risk profiles, demographic, socio-economic characteristics, biomarkers and molecular subpopulations, to arrive at the possible proposal of a “precision” treatment, specific to the individual patient so that this can benefit from the treatment itself by limiting the risk of toxicity related to it. All of this, in part, is alreadily applied in daily clinical practice in oncology, but it is still a challenge in radiotherapy (RT) treatments (3).

On the one hand, in fact, the field of radiation therapy has substantially evolved over the last decades; in particular technological advances have led to the development of various high-precision techniques such as modulated intensity radiotherapy (IMRT), radiotherapy guided by image (IGRT), stereotactic radiotherapy (SBRT), particle therapies and brachytherapy, which allow for delivering the radiation dose more accurately, by administering high doses to the tumor and limiting those to surrounding organs (4). In HNC this has resulted, from a clinical point of view, in the reduction of acute and late severe toxicities and in a better quality of life (3). Despite this, currently, RT treatments are planned on the anatomy of the individual patient but are not modulated according to the biological characteristics of that patient’s specific neoplasm; patients with tumors at the same stage and site are considered similar and then treated at the same way (5).

The implementation of precision medicine in oncology and, especially, in RT therefore requires a precise understanding of the behavior of the disease, even at the molecular level aiming at a biology-driven radiotherapy approach. In this context, the identification of prognostic and predictive factors is of considerable interest. Specifically, the prognostic factor is defined as that factor that describes the natural progression of the disease with or without a therapeutic intervention; by predictive factor, on the other hand, we define the factor that describes the response to a specific therapeutic regimen, so in fact it describes the response or absence of response (if we are referring to the effectiveness of the treatment) or the development of toxicity. In literature several data underline the importance and the potential impact of some biomarker; in radiation oncology those that have been shown to influence response to treatment are focused on some biological characteristics.

In this review we try to describe which novel prognostic and predictive factor in HNC are developed and which of those might be specific for evaluated radiation response in clinical practice (**Table 1**).

**Table 1:** Overview on the main points described in the review.

Potential biomarker		
Biological	Viral factors	
	Hypoxia	RSI
		GARD
Immunological	TILs	
	Treg cells	
Imaging	PET/CT	
	MRI	

RSI, radiosensitivity index; GARD, genomic-adjusted radiation dose; TILs, tumor infiltrating lymphocytes; PET/CT, positron emission tomography/computed tomography; MRI, magnetic resonance imaging.

## Biology Determinants

### EBV-DNA

Several data reported on patients with nasopharyngeal cancer show that pre-treatment plasma concentrations of EBV-DNA and the presence or absence of viral DNA in plasma after RT are statistically significant correlate with overall survival (2-year OS: 56.3% in patients with detectable EBV-DNA *vs* 96.7% in patients with undetectable EBV-DNA after RT) and with relapse-free survival (RFS) (6). Data revealed that several months before recurrence high serum EBV-DNA could be detected demonstrating its potential as a biomarker of subclinical disease. Moreover, the presence of high pre-treatment plasma viral levels also correlates with the risk of developing distant metastases (7). High post-treatment EBV-DNA is a recognized negative prognostic factor that a phase II–III trial has incorporated serum EBV-DNA to personalized treatment in stage II–IV nasopharyngeal cancer (NCT02135042). All the 924 planned patients of this randomized trial will first undergo concurrent chemo-radiotherapy and then are randomized according to plasma EBV-DNA. If plasma EBV-DNA is not detectable patients are randomized to standard adjuvant chemotherapy or observation. Instead if plasma EBV-DNA is detectable patients will be randomized to standard cisplatin and fluorouracil chemotherapy *versus* gemcitabine and paclitaxel (8).

### HPV Infection

Another particularly important example in oropharyngeal squamous cell carcinoma (OPSCC) is represented by HPV infection; it is widely demonstrated that patients with HPV-related head and neck neoplasia have better outcomes than HPV negative patients; specifically, HPV positivity is the strongest prognostic factor for survival (9). This factor is so significant that the classification of oropharyngeal tumors has been changed, initially leading to a classification into three risk groups (low, intermediate, and high) based on the combination with other parameters (HPV, smoking, and stage of disease) (9) and in 2018, the TNM staging was modified.

Considering these better outcomes, the identification of biomarkers of HPV driven disease is extremely important; actually p16 expression is considered a surrogate for HPV+ OPSCC; however, the association of p16 with HPV DNA positivity is more accurate in predicting recurrence free survival (10). Currently other factors as HPV oncoproteins and serum E6 and E7 proteins are under evaluation in order to documenting biologically active HPV infections rather than mere HPV DNA detection (11).

Some data suggest that HPV-driven OPSCC are associated with serum antibodies to the HPV oncoproteins E6 and E7; it seems that pre-treatment levels of these antibodies are related to disease-free survival in HPV+ OPSCC, indicating that a highly immunogenic response to these proteins before treatment can be detected (12). Moreover further data demonstrated that clearance of E6 and E7 antibodies is lower in patients who develop recurrence after chemoradiotherapy compared to those who do not recur; thus E6 and E7 antibodies might be potential biomarkers in HPV OPSCC (13). Considering the better outcome of HPV-positive OPSCC, a large number of trials are evaluating a de-escalation approach for this subset of patients; de-intensification strategies could be several and regard both chemotherapy and radiation therapy. Here we concentrated on RT approaches that could consider a reduction of RT doses or volumes. Several studies have been conducted and others are on-going to investigate this option: the ECOG1308 trial, a non-randomized phase II trial, enrolled 90 HPV16 and/or p16-positive, stage III–IV OPSCC patients. They received three cycles of induction chemotherapy (IC) with cisplatin, paclitaxel, and cetuximab and, according to IC response, received intensity modulated radiation therapy (IMRT) 54 Gy with cetuximab in case of a clinical complete response (cCR) or IMRT 69.3 Gy and cetuximab in those patients with less than cCR to IC.

The oncological outcomes were interesting with 2-year progression-free survival (PFS) and OS rates of 80 and 94% respectively, for patients with primary-site cCR treated with 54 Gy of radiation; the authors concluded de-intensification of treatment in selected patients with HPV related OPSCC could be explored in further study; moreover it should be considered that RT dose reduction improved significantly swallowing and nutritional status (14).

The Quarterback trial, a phase III non-inferiority randomized trial, evaluated the oncological results of reduced RT dose (56 *versus* 70 Gy concomitant to carboplatin) after induction chemotherapy (three cycles of TPF) in 20 HPV+ locally advanced OPSCC. The 3 yy PFS and the OS were 87.5% for standard CRT and 83.3% for reduced CRT for both endpoints, respectively (15). At the 2019 ASTRO annual meeting the preliminary results of NRG-HN002 trial were presented: it is a randomized phase II trial that compared accelerated IMRT (60 Gy in 5 weeks) *versus* IMRT (60 Gy in 6 weeks) plus weekly CDDP in p16+ OPSCC; both arms could be considered testing some form of de-escalation (the former provides omission of chemotherapy but accelerated RT, the last reduction in RT dose −60 Gy *versus* standard 70 Gy). It was designed to select the schedule that achieve acceptable PFS and swallowing function for further trial, this was met by IMRT plus CDDP arm (16).

Other two on-going phase II trials evaluate reduced RT doses associated with standard chemotherapy (NCT03215719–NCT01088802) in favorable risk HPV positive OPSCC (17, 18). Moreover another on-going trial is evaluating volume (level IB lymph node is excluded from the elective nodal volumes) and dose de-intensified RT (60 Gy to gross tumor volume and 54 Gy to region at risk of subclinical disease in 30 fractions) for p16+ squamous cell carcinoma of the oropharynx with CDDP based chemotherapy (19).

The biological rationale for these approaches is based on the fact that the best outcomes of HPV-positive OPSCC may be linked to response to radiation therapy. *In vitro* studies showed that HPV positive cells presented an increase in cell thickness and motility after RT; this suggests that they undergo apoptotic death and are more radiosensitive than HPV negative cells (20). These preclinical data are confirmed by retrospective analysis of several clinical trials. The critical point is that all patients with HPV-positive tumors and with defined clinical characteristics are treated in the same way. Although some results support de-escalation approaches, some concerns were raised as they can result in detrimental outcomes for patients (in RTOG 1016, a randomized non-inferiority trial that compared RT plus cetuximab *versus* RT plus cisplatin, a reduced LRC and OS with de-escalation was detected) (21, 22). This could probably be due to lack of adequate risk stratification system to identify the most suitable HPV+ patients for de-intensification trial; in fact the development of more accurate treatment response classifiers is needed. The next step that should be integrated is a more personalized approach based on the biology of the tumor that might help to develop criteria to better define lower risk HPV+ subpopulations. On this concept an interesting ongoing trial is a non-inferiority phase II study (NCT03323463) which randomized patients with HPV positive and hypoxia negative T1–2, N1–2c (AJCC, 7th ed) OPSCC to descalated RT associated with two cycles of chemotherapy *versus* standard chemoradiotherapy. RT schedule provides a total dose of 30 Gy in 3 weeks (2 Gy per fraction, 5 days per week), deliver to GTV, postoperative bed and all area at microscopic risk of disease. The estimated enrollment is of 300 patients (23). The most interesting point of this trial is that it is specific for patients with HPV+ but hypoxia negative tumors.

### Hypoxia

Hypoxia is another parameter widely studied in head and neck neoplasms; it is present in many solid tumors and is well known to be related to radioresistance and therefore indicative of an unfavorable prognosis. Several studies tried to identify biomarker of tumor hypoxia. The methods for detecting intratumoral hypoxia have continuously evolved over the years but their application in clinical practice is still difficult; more recently the attempt is to identify gene expression of hypoxia as endogenous biomarker (24, 25). Specifically, in HNC, gene expression signatures (a group of gene instead of a single one) were developed (26).

Toustrupt et al. identified a method for characterizing the hypoxic state of a tumor based on the quantification of hypoxia-specific genes within the tumor biopsy and generated a model that could improve the ability to individualize treatment according to this characterization. This classification, based on the evaluation of 15 hypoxia-sensitive genes assessed as the best to be able to discriminate between “plus” and “less” hypoxic HNC, was applied to 323 biopsies of patients enrolled in the DAHANCA 5 study (a clinical trial that randomized patients to placebo *versus* hypoxic modification with nimorazole plus RT). The classifier categorized 114 tumors as “more” hypoxic and 209 as “less” hypoxic. In the group “more” hypoxic, patients treated with nimorazole and RT presented a better locoregional control failure rate at 5 years when compared to patients treated with RT alone (79 *vs* 46%). No difference was detected between treatments in the group classified as “less” hypoxic suggesting that hypoxic modification of RT could be useful only in a subgroup of patients with gene expression classified “more” hypoxic tumors. Therefore, this 15-gene hypoxia classifier attains both prognostic and predictive potential (25).

### Radiosensitivity Index

Eschrich et al., considering the expression of 10 genes, developed a new model of intrinsic tumor radiosensitivity, to create the radiosensitivity index (RSI) that is directly proportional to tumor radioresistance. The lower is this index, the higher is the tumor’s radiosensitivity. The use of this model has been clinically validated on two separate cohorts of patients and subsequently in HNC patients treated with radical chemo-radiotherapy; what emerges is that, by classifying the tumors into “more” radiosensitive and “less” radiosensitive on the basis of the RSI, it is possible to distinguish subgroup of patients with different outcomes. In HNC the radiosensitive group presented an improved 2-year locoregional control (2-year LRC 86 *vs.* 61%, *p* = 0.05), underling that this model is able to identify biological similarities strictly linked to tumor radiosensitivity across disease sites (27). Another extremely interesting thing of this index is that seems to have a predictive value only in patients treated with RT, and not for other treatments as surgery or chemotherapy. Therefore, RSI can be considered an independent predictor of response to RT alone (27). Based on these data, the concept that the benefit of RT is not uniform in all patients and that it varies in genomically distinct subpopulations is increasingly evident.

### Genomic-Adjusted Radiation Dose

A further potential of this index, however, arises from its combination with linear-quadratic model to create the GARD “genomic-adjusted radiation dose”, a clinical model that represents a molecular estimate of the fraction of cells surviving at 2 Gy and could allow individualizing the dose of radiation therapy based on the radiosensitivity of the individual tumor. GARD model was prospectively validated by Scott et al. in 2017 and is associated with outcomes in five clinical cohorts. The GARD varies widely among the various tumor histotypes, a high GARD value provides a high therapeutic effect for RT. In fact, lower median GARD values for HPV negative oropharyngeal cancer and higher for HPV positive oropharynx have been detected, therefore in line with the superior clinical results that are normally obtained in HPV related OPSCC (28). GARD should not be considered a predictive factor but rather a tool that allows to personalized treatment on the specific tumor of the single patient. This could be obtained in different way as: 1—a modulation (increase or reduction) of radiation dose based on GARD value, 2—a combined treatment with drugs modifier of hypoxia, 3—change in treatment modality, for example in less radiosensitive tumors, GARD might suggest that RT dose required is beyond the tolerance of normal tissue so the patients might be treated with surgery/chemotherapy. Obviously, these represent hypothetic potentiality of GARD model, moreover it should be considered some limits of GARD as that it accounts only for tumor radiosensitivity and no other important biological parameters as proliferation, DNA repair or patients characteristics.

## Head and Neck and Immune System

The immune response to tumors is a recent studied factor; it is extremely complex with the involvement of different cell types both of the adaptive and innate immune systems, and it plays an important role in neoplastic progression (29). The immune system stimulates the elimination of tumor cells and the control of tumor growth; moreover, the tumor microenvironment is highly suppressive and hinders the physiological activity of T cells (29). Generally, HNC is considered a cold tumor capable of creating evasion effect and being less attacked by natural and adaptive immunity. In the context of head and neck neoplasms, patients with high T lymphocytes infiltrated (TILs) tumors appear to have better OS, PFS, and distant metastasis-free survival compared to patients with poorly infiltrated tumors (30, 31). The major correlation with oncological outcome has been demonstrated for CD8+ effector lymphocytes, in fact their rate is higher in HPV-positive cancers, and this could partially explain the better outcome of this subpopulation (32). The CD3 + and CD8 + TILs represent strong prognostic and also predictive markers capable of identifying a subset of HNC patients who have a greater probability of progression and shorter survival after radical chemoradiotherapy (30). The use of TILs as biomarkers to predict recurrence and death from cancer is very interesting especially in advanced diseases; in fact in this setting the implementation of immunotherapy combined with chemoradiotherapy could be particularly advantageous. This association could have a double effect, from one side immunotherapy can enhance the efficacy of RT as a locoregional treatment but RT could work also as an *in situ* vaccination to increase the efficacy of immunotherapy (33); in fact RT is able to upregulate programmed death ligand 1 (PD-L1) on tumor cells, which may increase the response to some immunotherapies.

Furthermore regulatory T cells (Tregs) have the role to suppress immune system and they act inhibiting the action of cytotoxic T cells (34). Several data demonstrated a higher Treg rate in peripheral blood of HNC patients compared to healthy controls, and it seems that patients with high Treg infiltrates cancer present a worse prognosis (35). Also dendritic cells play an important role T cell responses as they present tumor antigens on MHC-I; this leads to activation of CD4+ and CD8+ naïve T cells and to differentiate in effector T cells. Some data showed that tumor with high rate of dendritic cell tumor lead to better outcome in HNC patient (36).

## Role of Molecular Imaging in Research

Another strategy that has been explored is the integration of imaging into precision care. To date the 2-deoxy-2-[18F]fluoro-D-glucose (FDG) positron emission tomography/computed tomography (PET/CT) is a standard diagnostic methodic used in HNC patients for staging, restaging, RT planning, and outcome evaluation (37, 38). Several data published in literature underline how changes of neoplastic glucose metabolism, evaluated by FDG PET, are able to predict tumor response rates and oncological outcomes in several solid cancer (39). In particular as regards PET in HNC the major data are validated for SCCHN and nasopharyngeal cancer.

In this settings, PET/CT could help radiation oncologist for target delineation and in radiation planning (39). Moreover, it has been suggested that FDG is able to detect primary tumor site in about 25% of patients with unknown disease (40) and at baseline, it allows quantification of the tumor tracer uptake thus producing semiquantitative values that can be indicative of prognosis. The prognostic role of these parameters in HNC is under active investigation.

In fact, the possibility to identify pre-treatment biomarkers correlated with outcome could be of particular interest in some subgroups of patients with the aim to intensify treatment (41). The most widely used semiquantitative parameter in oncology is the so-called maximum standardized uptake value (SUVmax). The SUV is a semiquantitative measure of the tracer uptake in a region of interest that normalizes the lesion activity to the injected activity and a measure of the volume of distribution (usually total body weight or lean body mass). Some studies of patients with HNC have shown no predictive value (42, 43), while others have suggested a potential prognostic significance of SUVmax of the primary lesion is (44, 45).

Of note, SUV and SUVmax lack of reproducibly between different PET scanners (also in terms of its uptake time dependence) and thus might be not suitable for the multicenter research settings (46).

It has been suggested that the use of uptake time normalized tumor-to-blood SUV ratio (standardized uptake ratio, SUR) might remove most of these shortcomings improving test–retest stability and providing significantly better prognostic value compared to tumor SUV (39). However, more recently, other PET-based parameters have emerged as potentially valuable for the prognostic stratification in several oncologic diseases including HNC. In particular data are already available with respect to the predictive value of metabolic tumor volume (MTV) and total lesion glycolysis (TLG) (47). MTV is defined as the sum of the volume of voxels with SUV exceeding a certain threshold value in a tumor, reflecting the volume of FDG activity in a tumor assessed by automated volume of interest delineation, while TLG is obtained by multiplying MTV and the mean SUV of the MTV. Romesser et al. found in patients treated and submitted to chemoradiation that a lower MTV is related to improved local control, PFS and OS (42). Similarly, Hanamoto and colleagues found that high metabolic burden in terms of TLG and MTV can independently predict treatment failure more accurately than SUVmax or SUVmean (48). Suzuki and colleagues demonstrated that a TLG ≥5.4 was significantly linked with shorter disease-specific survival, distant metastasis-free survival, and lung metastasis-free survival in laryngeal or pharyngeal cancer patients treated with salvage surgery (49). Finally, recent systematic reviews (one of them including also a meta-analytic analysis) evaluated the relationship between semiquantitative metabolic parameters and outcomes of patients with HNC. These data demonstrated that higher pre-treatment MTV is linked to worse OS, PSF, and locoregional control (50, 51).

Moreover the distribution of FDG uptake in tumors has been studied by other groups as potential prognostic factor of complex heterogeneity parameters such as entropy or textures (52). In particular, Meyer et al. evaluated the relationships between histogram analysis of apparent diffusion coefficient (ADC) values and FDG PET-derived parameters in SCCHN (53). In fact, also functional imaging MRI, such as diffusion-weighted imaging (DWI) can be added to provide further insight into tumor microstructure (54). DWI measures random water movement and can be quantified by the ADC. Previously, various studies identified an inverse relationship between ADC values, cellularity, and proliferation thus suggesting that ADC values mirror tumor microstructure (55). Meyer et al. showed that entropy derived from ADC maps is strongly associated with MTV and TLG in HNC (53). This correlation demonstrated to be stronger in G1/2 tumors and entropy; SUVmax, SUVmean, TLG, and MTV were statistically significantly higher in T3/4 tumors in comparison to T1/2 carcinomas (53). Some trials compared MRI to PET/CT with the aim to evaluate advantages and disadvantages of both methods. Cao et al., in 54 patients with locally advanced SCCHN, investigated p16+ effects on imaging parameters, differences between imaging biomarkers of tumors with local, regional or distant progression and the predictive values of MRI and PET biomarkers. They found that the p16− primary tumors had elevated ADC values pre-RT and low early response rates compared to p16+ tumors; also, high mean apparent diffusion coefficient (ADC) value pre-RT is a hazard for local and regional failure of p16− tumors. Moreover multiple MRI and PET imaging parameters predicted regional and distant failure, but the nodal GTV defined on anatomic MRI was the strongest predictor. They concluded that the performance of MRI related parameters is stronger than PET parameters and that MRI could play an important role from treatment planning, to early response assessment, and boost target definition with different but complementary information (56). Moreover, a comparison between MRI and PET/CT was done by Wong et al. in predicting the response to definitive treatment after induction chemotherapy in locally advanced SCCHN. Firstly, they detected that changes in functional and molecular imaging parameters with both modalities after first induction chemotherapy cycle are representative of its full effects. They did not find pre-treatment ADC to be predictive of treatment outcome; this datum, that is partially in contradiction with other data literature, is explained by authors with the high prevalence of HPV related tumors in their cohort of patients, as HPV related OPSCC often presented unique histologic features as micronecrosis (57). Instead in the context of radical chemo-radiotherapy, increase in ADC has been demonstrated in responders and a lower increase or decrease 1–3 weeks into radiotherapy in non-responders (58, 59). To conclude both PET/CT than MRI could give complementary information to try to identify patients with better or worse prognosis.

Besides the role in the potential intensification of therapy, FDG PET might also have a role in therapy de-intensification. In patients with HNC, viral-related mechanisms have a relevant role in developing a robust personalized medicine associated with specific tumor characteristics at individual level thus guiding appropriate treatment selection. In particular, HPV and EBV provide robust prognostic biomarkers in SCCHN and nasopharyngeal carcinoma respectively and are now being incorporated into clinical trials (60). In this setting FDG PET might help to categorize patients according to risk of recurrence and then to tailor treatment. Floberg et al. in 153 HPV related OPSCC patients demonstrated that the optimum MTV (identified as 24 cm^3^) is significantly related to freedom from recurrence, freedom from distant metastasis and OS; these data support the use of MTV as prognostic marker in patients treated with surgery as well as definitive radiotherapy (61).

Another parameter that has been investigated is the heteroneity index (HI) that is a quantitative measure of the intratumoral heterogeneity of 18F-FDG uptake although HI has been reported as a prognostic factor in locally advanced nasopharyngeal carcinoma (62).

Moreover Kimura et al. evaluated HI in patients with oral cavity SCC and showed that it is a statistically significant prognostic factor for OS in patients with OSCC treated with primary surgery (63). Finally, although 18F-FDG is by far the most frequently used radiopharmaceutical in HNC, glycolysis is not the only metabolic process or biochemical pathway that can be visualized.

In particular, PET technology is able to track the presence of tumor hypoxia (39). As mentioned above, tumor hypoxia is related with worse outcomes and is a major driver in treatment resistance (39, 64). In fact the possibility to track hypoxia *in vivo* is of interest to guide the use of hypoxia sensitizers or of specific IMRT. Most studied evaluated [18F]Fluoromisonidazole (18F-FMISO) PET/CT as it was the first hypoxia tracer (39, 65); it is a 2-nitroimidazole molecule and is well known that imidazole derivates are trapped in hypoxic cells. Studies aiming to evaluate features of patients’ non-responders to RT by means of 18F-FMISO PET have suggested that around a half of locoregional failures in SCCHN occur because of hypoxia. Despite this 18F-FMISO is not normally used in the clinical practice, this is due mainly to its high lipophilicity and slow clearance from normal tissues (39) with subsequent difficult in hypoxia identification. Another imidazole derived tracer is [18F] Fluoroazomycin Arabinoside (FAZA) that is characterized by a faster clearance from blood and non-target tissues (39).

Finally, the emerging role of immunotherapy and in particular of immune checkpoints inhibitors (ICPIs) in several cancers, including HNC have further stimulated the development of PET-based prognostic biomarkers (66). RT can modify the expression of some receptor (as PD1/PD-L1) on cancer cells or on myeloid cells, which may affect response to PD-1-based immunotherapy. On these basis novel and promising non-FDG PET tracers have been developed with the aim of predicting response to ICPIs (67, 68).

In fact, PD-L1 expression by immunohistochemistry has been correlated with response and survival following PD-(L)1 monoclonal antibody therapy. However, a lack of response has also been demonstrated in patients with PD-L1 expression and has been linked to heterogeneity of PD-L1 expression within tumors. PET studies in preclinical models have tested this hypothesis.

Some preliminary data on PET imaging with 18F-BMS-986192, 89Zr-Nivolumab, and 89Zr atezolizumab performed in patients with different tumor types before treatment with ICPIs detected a tumor tracer uptake heterogeneity in different patients and in different lesions of the same patient (67, 68).

To conclude both PET/CT could give complementary information to try to identify patients with better or worse prognosis than MRI.

A prognostic effectiveness of baseline FDG PET parameters as biomarkers of OS, DFS, and DM among patients with HNC has increasingly been recognized. Parameters (MTV and TLG) measuring metabolic tumor burden seemed relevant for identifying patients with a higher risk of treatment failure and early disease progression. However, further large-scale studies including patients stratified according to localization and further analysis of the textural indices are required to define a reliable FDG PET-based prognostic model of mortality and recurrence risk for HNC patients. Finally, while FDG remains by far the most frequently used radiopharmaceutical in HNC, novel PET radiotracers especially tracking hypoxia as well as immunoPET imaging might be of further value in the baseline and early prognostic stratification of HNC.

## Conclusions

At present only HPV can be considered a novel prognostic biomarker and must be used in clinical practice to stratify patients among those with better or worse prognosis. The role of the HPV should be integrated with other parameters related to the patient and the tumor that modulate the outcome of the treatments. Unfortunately, even if HPV is the strongest available biomarker, it does not yet allow us to modify the treatment approach outside clinical trials.

Moreover, a prognostic effectiveness of baseline FDG PET parameters as biomarkers of OS, DFS, and DM among patients with HNC has increasingly been recognized.

Despite the increase in the number of studies that investigate possible predictive biomarker in HNC, currently there are no biomarkers of response to RT used as standard of care in clinical practice; certainly some are promising, in particular to understand when to use innovative immunological drugs associated with RT, but further evaluations are needed.

## Author Contributions

All authors contributed to the study conception and design. LB and SM wrote the paper and reviewed the final manuscript. RC reviewed and edited the final manuscript. All authors contributed to the article and approved the submitted version.

## Conflict of Interest

The authors declare that the research was conducted in the absence of any commercial or financial relationships that could be construed as a potential conflict of interest.
